# Cohesin promotes HSV-1 lytic transcription by facilitating the binding of RNA Pol II on viral genes

**DOI:** 10.1186/s12985-021-01495-2

**Published:** 2021-01-23

**Authors:** Xin Li, Yafen Yu, Fengchao Lang, Guijun Chen, Erlin Wang, Lihong Li, Zhuoran Li, Liping Yang, Xia Cao, Nigel W. Fraser, Jumin Zhou

**Affiliations:** 1 Key Laboratory of Animal Models and Human Disease Mechanisms of Chinese Academy of Sciences/Key Laboratory of Healthy Aging Research of Yunnan Province, Kunming Institute of Zoology, Kunming, 650223 Yunnan China; 2 Institute of Health Sciences, Anhui University, Hefei, 230601 Anhui China; 3 Key Laboratory of Second Affiliated Hospital of Kunming Medical University, Kunming, 650000 Yunnan China; 4 Department of Microbiology, Perelman School of Medicine, University of Pennsylvania, Philadelphia, PA 19104 USA

**Keywords:** Cohesin, SMC1, Rad21, HSV-1, Transcription

## Abstract

**Background:**

Herpes Simplex Virus type I (HSV-1) is a large double-stranded DNA virus that enters productive infection in epithelial cells and reorganizes the host nucleus. Cohesin, a major constituent of interphase and mitotic chromosomes comprised of SMC1, SMC3, and SCC1 (Mcd1/Rad21), SCC3 (SA1/SA2), have diverse functions, including sister chromatid cohesion, DNA double-stranded breaks repair, and transcriptional control. Little is known about the role of cohesin in HSV-1 lytic infection.

**Methods:**

We measured the effect on HSV-1 transcription, genome copy number, and viral titer by depleting cohesin components SMC1 or Rad21 using RNAi, followed by immunofluorescence, qPCR, and ChIP experiments to gain insight into cohesin's function in HSV-1 transcription and replication.

**Results:**

Here, we report that cohesion subunits SMC1 and Rad21 are recruited to the lytic HSV-1 replication compartment. The knockdown results in decreased viral transcription, protein expression, and maturation of viral replication compartments. SMC1 and Rad21 knockdown leads to the reduced overall RNA pol II occupancy level but increased RNA pol II ser5 phosphorylation binding on viral genes. Consistent with this, the knockdown increased H3K27me3 modification on these genes.

**Conclusions:**

These results suggest that cohesin facilitates HSV-1 lytic transcription by promoting RNA Pol II transcription activity and preventing chromatin's silencing on the viral genome.

## Background

The herpes family of DNA viruses can be divided into neurotropic or alpha Herpes viruses such as HSV and VZV, which latently infect sensory neurons, and lymphotropic Herpes viruses including EBV, KSHV, and CMV, which form latent infections in lymphoid cells, Initial Herpes virus infection begins in epithelial cells before transferring to the cell types which are destined to become latently infected [1]. Herpes Simplex Virus type I (HSV-1) is a linear double-stranded DNA virus with a large 152 kb genome, coding about 80 genes. It enters productive infection in epithelial cells and can establish latent infection in ganglia sensory neurons as a non-integrated, nucleosome-associated episome to colonize the host nucleus. During lytic infection, HSV-1 assembles its chromatin and synthesizes, sequentially, viral immediate-early protein (IE), early protein (E), and late proteins (L). The resulting productive infection of HSV-1 causes oral herpes, viral keratitis, and genital herpes, a serious condition affecting 18% of the adults in the US.

Cellular RNA polymerase II is responsible for viral gene transcription and the CTD ser5P (C-terminal domain ser5 phosphorylated) modified form is vital in transcriptional regulation [2–4]. Host chromatin assembly factors, host histones, and histone variants are also utilized to assemble HSV-1 chromatin [5–7]. The latent Herpes virus genomes are generally considered to be packed into chromatin in similar ways to the host chromatin [8, 9], which are composed of closely packed, modified histones including H3K27me3 and H3K9me3.

Cohesin complex comprises SMC1, SMC3, SCC1/Rad21, and SA1/2, and is essential for chromatid cohesion and segregation processes [10, 11]. Besides its defining activity of mediating sister chromatid cohesion, cohesin is also important for DNA double-strand break repair, transcriptional control, and long-range chromosomal interactions [10]. Cohesin is also implicated in the interaction between the virus and host cells, where it interacts with or regulates several DNA viruses, including EBV, KSHV, and HPV [12–14]. For example, Rad21 maintains KSHV latency; its deletion or cleavage leads to KSHV lytic infection from latent infection in KSHV-positive pleural effusion lymphoma cells [15]. Cohesin, but not CTCF, represses KSHV lytic gene activation during latency infection [16]. During KSHV lytic infection, cohesin and CTCF promote viral transcription initially but subsequently inhibit KSHV lytic transcription. In EBV, CTCF and cohesin are highly enriched in the LMP1 and LMP2A genes and promote their transcription through epigenetic regulation [17]. However, the function of cohesin in HSV infection has not been explored.

We investigated the role of two core cohesin subunits, SMC1 and Rad21, in HSV-1 lytic infection and found that cohesin is recruited to the HSV-1 replication compartment, its knockdown repressed the formation of the replication compartment, suggesting that cohesin promotes the formation of the HSV-1 replication compartment. Further analyses revealed that cohesin promotes HSV-1 lytic gene transcription, as the knockdown of SMC1 and Rad21 resulted in decreased immediate-early and late gene expression. Consistently, SMC1 and Rad21 knockdown caused a reduction in the viral genome copy number and viral yield. We further demonstrated that cohesin knockdown decreases RNA pol II occupancy at the lytic genes but increases RNA pol II CTD phosphor ser5 occupancy ratio. At chromatin level, SMC1 and Rad21 knockdown induced an elevation of the H3K27me3 enrichment to the HSV-1 genome. These results suggest that cohesin facilitates viral replication and transcription by promoting RNA Pol II recruitment to viral genes.

## Results

### Cohesin components SMC1, SMC3, and Rad21 are recruited by HSV-1 replication compartments

**Fig. 1:**
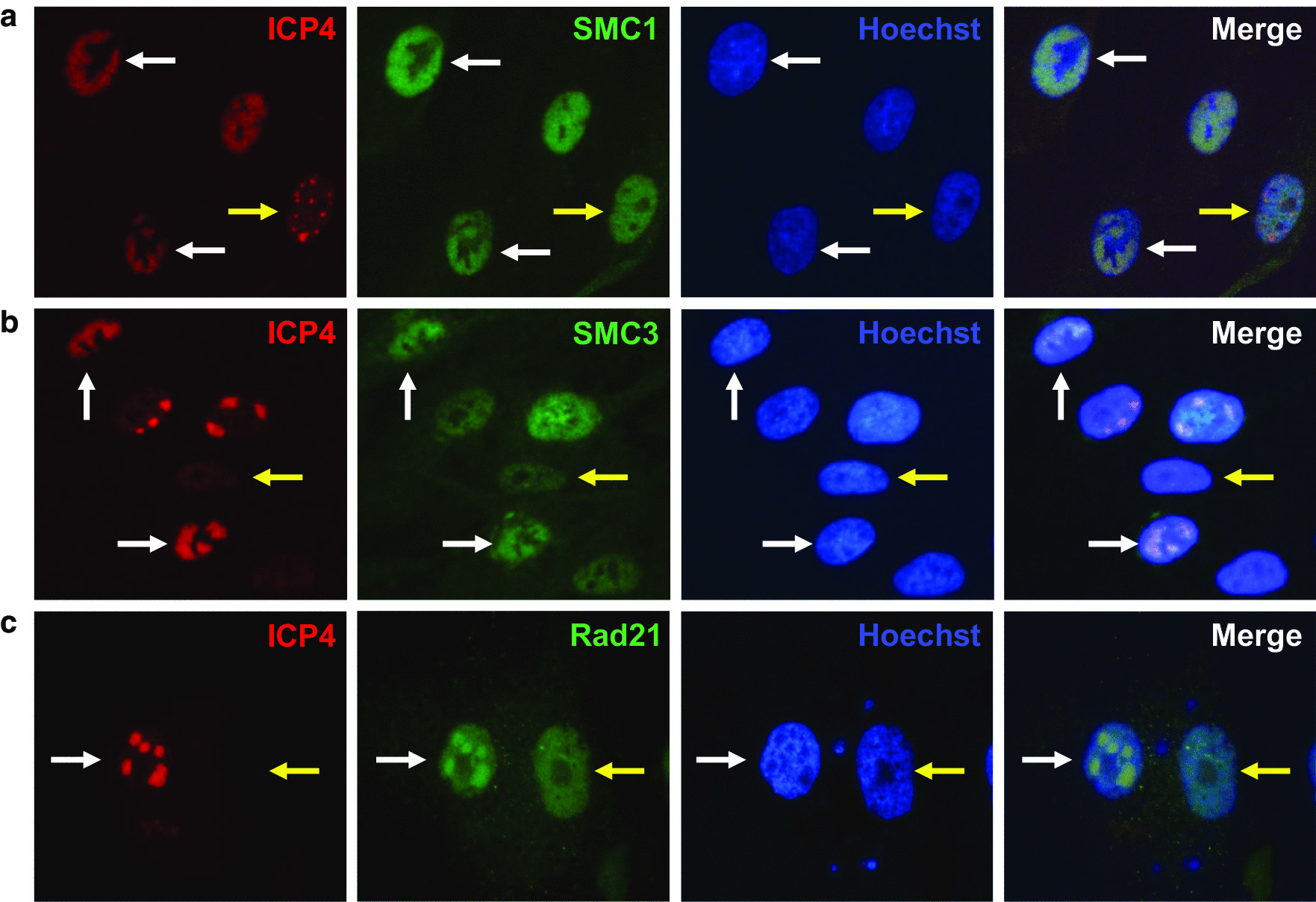
Cohesin components co-localize with HSV-1 replication compartments during viral lytic infection. BJ cells were infected with HSV-1 17+ for 5.5 h and were stained with antibodies against HSV-1 immediate-early protein ICP4 (red) and antibody against Cohesin components (green)-SMC1 (**a**), SMC3 (**b**), and Rad21 (**c**)

To determine whether the replicating HSV-1 genomes interact with the Cohesin complex, we first did double immunostaining with antibodies against the viral protein ICP4 to label the viral replication compartments and antibodies against SMC1, SMC3, and Rad21 in human primary fibroblast BJ cells at 6 h post-infection, a time point when HSV-1 replication centers are well organized. We found that all the cohesin components tested, SMC1, SMC3, and Rad21, were recruited to the HSV-1 replication compartments marked by ICP4 (Fig. 1). SMC1, SMC3, and Rad21 do not form obvious aggregated foci when there are no or few small HSV-1 replication compartments (Fig. 1a–c yellow arrow). SMC1, SMC3, and Rad21 were fully overlapped with ICP4 when the HSV-1 formed well-developed viral compartments (Fig. 1a–c white arrow). This data demonstrates that cohesin was recruited to the HSV-1 replication compartment and suggested that cohesin may play a role in HSV-1 infection.

### Cohesin knockdown leads to deficient HSV replication compartment formation

**Fig. 2:**
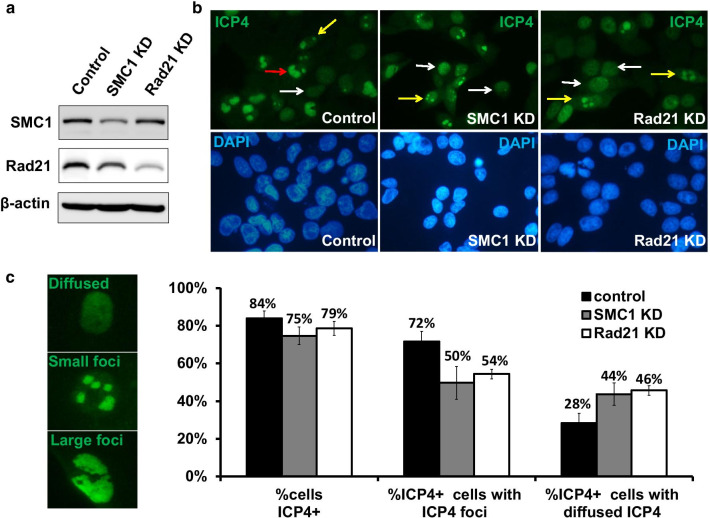
The depletion of Cohesin components inhibits HSV-1 replication compartments formation. **a** Western blot results show the efficiency of SMC1 and Rad21 knockdown by shRNA in BJ cells. **b** BJ cells were infected with HSV-1 at an MOI 5 for 5.5 h. Immunofluorescence results show the effects of SMC1 and Rad21 knockdown on HSV-1 replication compartment formation. **c** ICP4 staining patterns were defined as diffused, small foci, and large foci (left panel) to quantify the effects of SMC1 and Rad21 know-downs. The right panel shows the quantification analysis of ICP4 patterns before and after the Cohesin components knockdown

The number and size of viral replication compartments in the infected nucleus and the percentage of cells that develop these compartments are indicators of productive infection. To investigate whether cohesin plays a role in viral replication, we measured SMC1 and Rad21 knockdown effects on viral replication compartment formation in BJ cells. The effects of lentivirus-mediated knockdown in BJ cells are shown in Fig. 2a. Control and knockdown cells were infected with HSV-1 for 6 h at an MOI of 5. Knockdown of SMC1 or Rad21 by shRNAs led to a higher proportion of cells displaying diffused foci marked by IE gene ICP4 at 6 hpi (Fig. 2b, white arrow and yellow arrow), while HSV-1 tends to form large and well-developed compartment in control cells (Fig. 2b, red arrow and yellow arrow). Figure 2c (left) shows examples where SMC1 or Rad21 knockdown exhibited a disruption of foci structure, cells with small foci and matured foci as labeled by ICP4. We quantified the effects of knockdown by counting the percentage of ICP4 foci forming cells and percentage of ICP4 positive but diffused stained cells (Fig. 2b, c), and found that the knockdown of SMC1 or Rad21 moderately affected HSV-1 infection efficiency indicated by the percentage of ICP4 positive cells (84% in control cells, 75% in SMC1 knockdown cells and 79% in Rad21 knockdown cells). However, knockdown of SMC1 or Rad21 impaired the formation of HSV replication compartments as measured by ICP4 foci from 72% in control cells to an average of 50% in SMC1 knockdown cells and 54% in Rad21 knockdown cells (*P* < 0.05). Consistently, there is a corresponding increase of ICP4 positive, foci negative cells, 28% in control cells, 44% and 46% in SMC1 and Rad21 knockdowns, respectively (*P* < 0.05, Fig. 2c). The knockdown led to a failure of HSV-1 replication center formation in BJ cells, even though ICP4 protein was expressed. The viral replication compartment maturation from diffused status to obvious foci was faster in negative control cells than cohesin knockdown cells, suggesting that cohesin promotes the formation of the HSV-1 replication center.

### Cohesin knockdown led to decreased virus yield

**Fig. 3:**
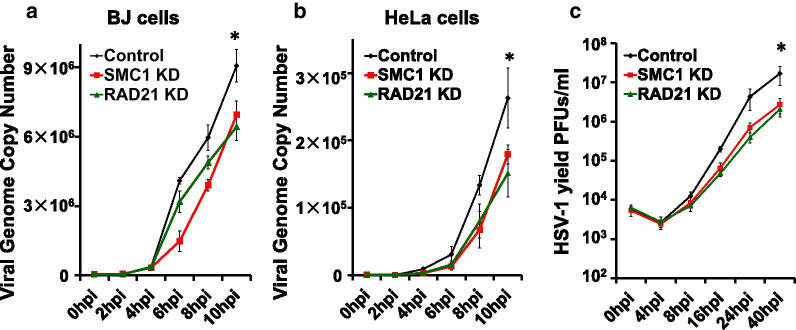
The depletion of Cohesin components affects HSV-1 production. **a**, **b** Human primary fibroblast BJ cells were infected with lentivirus containing control shRNA or shRNA targeting SMC1 or Rad21 for 48 h (**a**). HeLa cells were transfected with control siRNA or siRNA targeting SMC1 or Rad21 for 48 h (**b**). Then cells were infected with HSV-1 at an MOI 5. Genome copy numbers were detected at 0, 2, 4, 6, 8, and 10 h post-infection (hpi). Shown is DNA fold-change over control at 0 hpi. Error bars represent the standard deviation from three independent experiments. **c** HeLa cells were first knock-down SMC1 or Rad21 with siRNA, then were infected with HSV-1 at an MOI 0.1 and harvested cells and virus at various times post-infection. The viral yield was determined by plaque assay on Vero cells. *P < 0.05

Since cohesin is recruited to the HSV-1 replication compartment and facilitates the formation of replication centers, we investigated the role of cohesin in HSV-1 replication. We knocked down SMC1 or Rad21 in BJ and HeLa cells, followed by HSV-1 infection at an MOI of 0.1, and measured HSV-1 genome replication at a series of time points within 10 h post-infection through monitoring the viral genome copy number with qPCR. We found that as early as 6 hpi, viral copy number from cells depleted of SMC1 or Rad21 was less than from control cells, and by 10 hpi, the viral DNA copy number in knockdown cells was about twofold lower than that in control cells (Fig. 3a, b). The effects of knockdown of SMC1 and Rad21 were similar in both BJ cells and HeLa cells. These results suggest that cohesin is important for viral replication and growth. We further investigated HSV-1 titer in SMC1 and Rad21 knockdown HeLa cells for up to 40 h. We found that the viral production was significantly lower, up to an order of magnitude in SMC1 and Rad21 knockdown cells 16 h after initial infection (Fig. 3c). These results demonstrated that cohesin is a vital factor in supporting HSV-1 infection.

### Cohesin promotes HSV-1 transcription during lytic infection

**Fig. 4:**
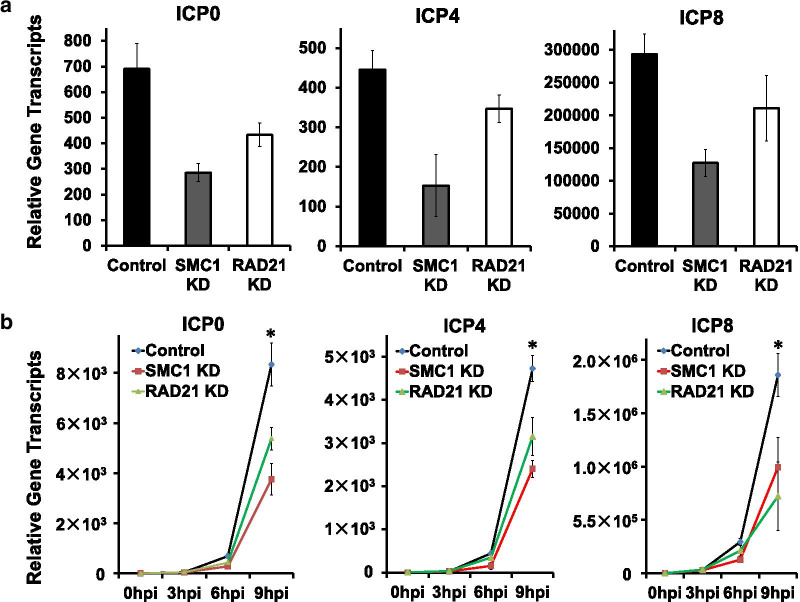
Effects of Cohesin components depletion on HSV-1 the immediate-early and early gene expression. **a** BJ cells were infected with HSV-1 at an MOI 5 after SMC1 or Rad21 knockdown by shRNA. ICP0, ICP4, and ICP8 gene transcripts were detected by RT-qPCR at 6 h post-infection (hpi). Shown is mRNA fold-change over control at 0 hpi. Error bars represent the standard deviation from three independent experiments. **b** HeLa cells were transfected with control siRNA or siRNA targeting SMC1 or Rad21 for 48 h. These cells were then infected with HSV-1 at an MOI 5. ICP0, ICP4, and ICP8 gene transcripts were detected by RT-qPCR at 0, 3, 6, and 9 h post-infection (hpi). *P < 0.05

To investigate how cohesin affects HSV-1 replication compartment development and viral transcription, we analyzed the effects of SMC1 and Rad21 knockdown on the transcription of viral genes representing each kinetic class (immediate-early, early, and late) including ICP0, ICP4, and ICP8 at 6 h post-infection in human primary fibroblast BJ cells. We found decreased RNA levels of viral genes from all three classes after the knockdown through RT-qPCR (Fig. 4b). A time-course experiment of these genes at 0, 3, 6, and 9 hpi in HeLa cells also showed consistent results with dramatic decreases in the expression of ICP0, ICP4, and ICP8 after the knockdown (Fig. 4a). These data demonstrated that cohesin plays an important role in regulating viral gene transcription and is thus involved in virus replication and the formation of the transcription/replication compartments.

### Cohesin and CTCF are recruited independently to the HSV-1 replication compartment

**Fig. 5:**
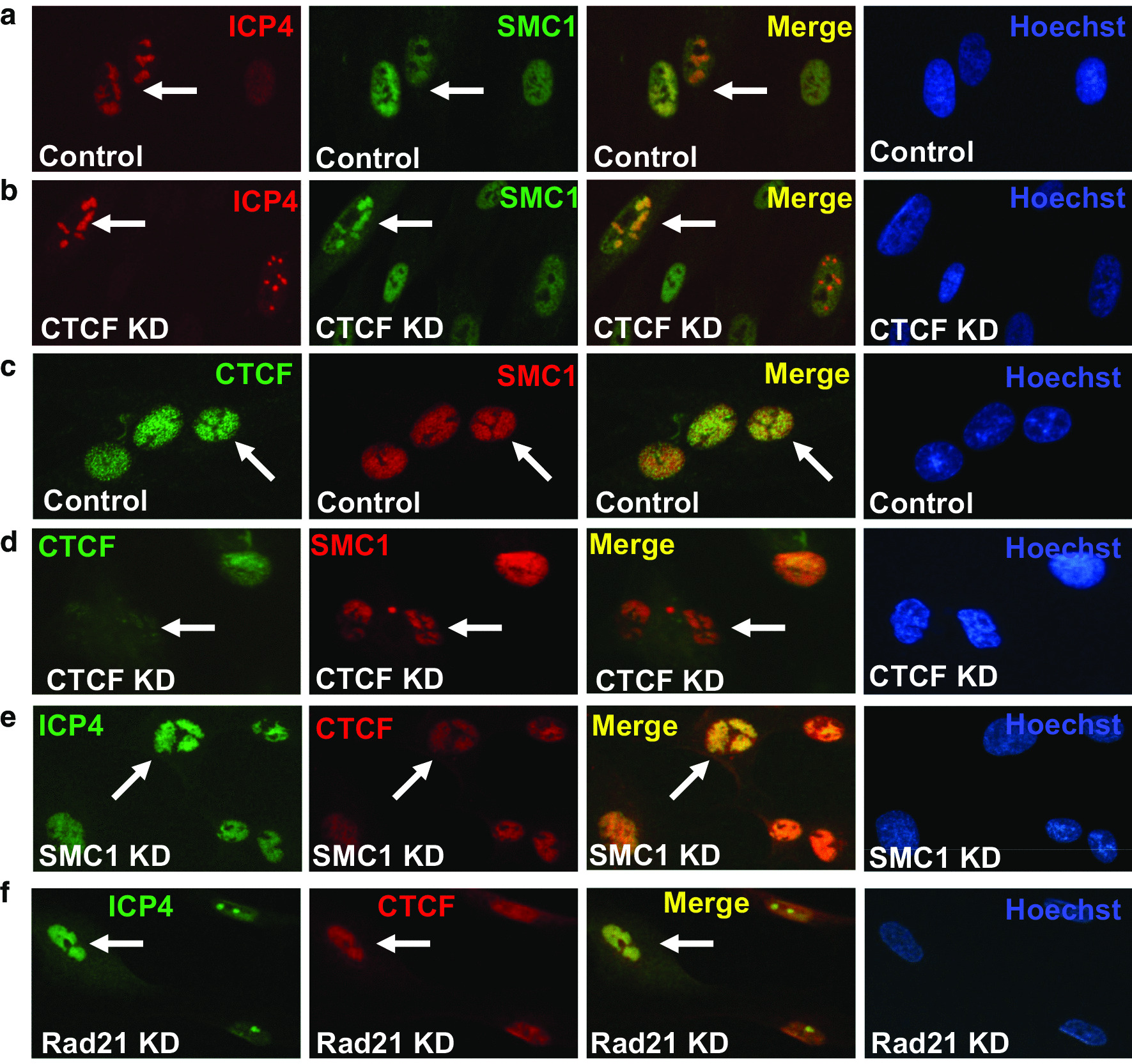
The recruitment of Cohesin components by HSV-1 is independent of CTCF recruitment. BJ cells were infected with lentivirus containing control shRNA or shRNA targeting SMC1 or Rad21 for 48 h. Then cells were infected with HSV-1 17+ for 5.5 h and were stained with antibodies against ICP4, CTCF, SMC1, SMC3, and Rad21

CTCF is an essential chromosome organizer with many essential functions in the nucleus [18–20]. Previously we found that CTCF can be recruited to HSV-1 replication compartments and plays an important in regulating HSV-1 transcription and replication [34, 35]. Cohesin and CTCF often cooperate in organizing the cellular chromatin structure and regulating gene expression [21, 22]. Cohesin relies on CTCF to bind to many genome loci for its transcriptional function, and blocking of CTCF binding can result in a loss of cohesin recruitment [23]. Since we found a role for cohesin in HSV-1 gene expression and viral growth, we wondered whether the recruitment of cohesin to HSV-1 replication compartments depends on CTCF. To test this, we did CTCF knockdown in BJ cells for 48 h and then subjected the cells to HSV-1 infection for 6 h at an MOI of 5. ICP4 was stained to monitor the infection of HSV-1. We found that SMC1 was recruited to ICP4 foci and can perfectly overlap with CTCF in the presence of HSV-1 infection (Fig. 5a, c), and this recruitment was unaffected by CTCF knockdown (Fig. 5b, d). Next, we did the reciprocal experiment to test whether cohesin knockdown affects CTCF recruitment to the HSV-1 replication compartment. Here, SMC1 or Rad21 was knocked down in BJ cells for 48 h and then subjected to HSV-1 infection for 6 h at an MOI of 5. We found that CTCF was still recruited to ICP4 foci in the presence of HSV-1 infection after SMC1 or Rad21 knocked down (Fig. 5e, f). These results suggested that cohesion and CTCF were recruited independently to the HSV-1 replication compartment.

### Cohesin knockdown led to decreased RNA Pol II recruitment and increased H3K27me3 binding to HSV-1 genes

**Fig. 6:**
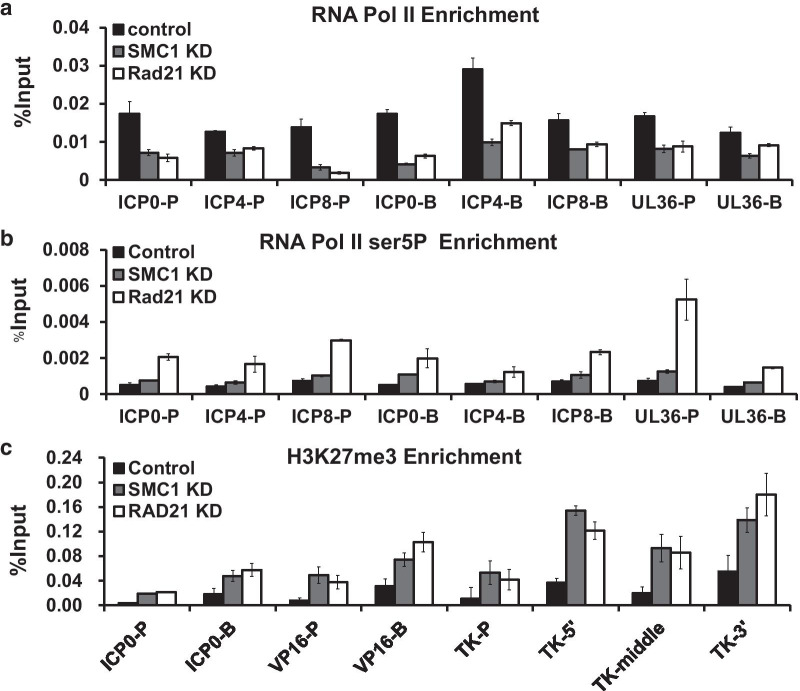
Cohesin components knockdown led to an overall decrease of RNA pol II binding and an increase of H3K27me3 binding to the HSV-1 genome. HeLa cells were transfected with control siRNA or siRNA targeting SMC1 or Rad21 for 48 h. Then cells were infected with HSV-1 at an MOI 5. The occupancy of RNA Polymerase II (**a**), RNA Polymerase II Phosphorylated on Serine 5 (**b**), and H3K27me3 (**c**) on both HSV-1 gene promoters (-P) and bodies (-B) were detected by ChIP-qPCR. Error bars represent the standard deviation from three independent experiments

The fact that cohesin knockdown leading to a large decrease in HSV-1 gene expression indicates that cohesin acts at the transcriptional level to support viral growth. Therefore, we tested the effect of cohesin knockdown on the binding of RNA pol II to the HSV-1 genome using chromatin immunoprecipitation (ChIP) and qPCR analyses. As demonstrated in Fig. 6a, SMC1 or Rad21 knockdown decreases the overall RNA pol II occupancy at the lytic genes, including ICP0, ICP4, ICP8, and UL36, suggesting that cohesin promote HSV-1 transcription by facilitating RNA Pol II recruitment (Fig. 6a). The large subunit of RNA Pol II has a 7 residue peptide repeat CTD, where serine 5 within these repeats could be phosphorylated by CDK7 while serine 2 could be phosphorylated by CDK9 [24]. Molecular and genome-wide analyses suggested that the Ser5P form (RNA pol II ser5P) represents pol II pausing, while Ser2P represents elongating form [24]. The composition of these modified forms of RNA Pol II at gene promoters indicates transcriptional activity. To test whether cohesin also affects the activity of total RNAP II and modification of RNA pol II at viral genes, we monitored the enrichment of total RNAP II and RNA pol II ser5P by ChIP-qPCR after SMC1 or Rad21 knockdown and found that the knockdown reduced the enrichment of total RNAP II while at the same time, increased the RNA pol II CTD phosphor ser5 occupancy (Fig. 6b). The reduction of total RNAP II and a concomitant increase of RNA pol II ser5P binding to the upstream of viral gene promoters and coding sequences in cohesin knockdown cells suggest that cohesin prevent the deposition of the paused form of RNA pol II on viral genes and, consequently, facilitate the transcriptionally active form of RNA Pol II binding to viral genes.

This result also suggests that cohesin could prevent the formation of silenced chromatin at viral genes. To determine whether cohesin also restricted the occupancy of non-permissive chromatin on the viral genome, we analyzed the effect of cohesin depletion on forming the polycomb-associated H3K27me3 modification on the HSV1 genome during viral infection. The ChIP-qPCR results showed that SMC1 or Rad21 knockdown increases the overall H3K27me3 modification at the viral genes, including ICP0, ICP8, VP16, and thymidine kinase (TK) (Fig. 6c). And these results indicated that cohesin could regulate the interaction between the host cell and HSV-1 by controlling the state of the HSV-1 chromosome.

## Discussion

Here we demonstrated that cohesin components SMC1, SMC3, and Rad21 were recruited to the HSV-1 replication compartment. SMC1 and Rad21 could promote HSV-1 replication compartment development at the early infection stage, and the knockdown of SMC1 and Rad21 resulted in a decrease of HSV-1 replication and viral copy number. SMC1 and Rad21 facilitated viral gene transcription by increasing the recruitment of overall level RNA Pol II and by preventing the enrichment of silenced chromatin mark H3K27me3. These results suggest that cohesin promotes HSV-1 lytic infection through facilitating viral replication compartment development and viral transcription.

Cohesin mediates sister chromatid cohesion by forming a ring structure during mitosis [25] and regulate gene transcription through forming long-range loops at many loci [26–28]. Cohesin shares many binding sites with a CTCF across the whole genome [29, 30], and together, they could function as boundary elements and form large chromatin domains [31]. Cohesin and CTCF co-localize in the latent transcript region within the KSHV genome and regulate lytic K14/ORF74 transcript [32, 33]. They also bind similar sites in the EBV latency membrane proteins LMP-1 and LMP-2A regions and are important for DNA loop formation with the origin of plasmid replication (OriP) enhancer [17]. We and others found that CTCF is recruited to the HSV-1 genome at multiple binding sites and localizes to a substructure within the viral replication compartments [34–38]. Here we found that cohesin components SMC1, SMC3, and Rad21 were also recruited to the HSV replication compartment, and this result is consistent with iPOND (Isolation of proteins on nascent DNA) data from others, which showed that cohesin interacted with replicating HSV-1 genome [39]. In addition to a direct role in transcription, cohesion could indirectly promote viral growth by participating in the DNA repair process and by chromatin organization. Since cohesin participates in DNA repair processes [40], it could help the virus during replication and resolve replication stress and DNA damage to the viral genome, inhibiting viral transcription and replication. The independent recruitment of CTCF and cohesion to the viral replication compartment supports this possibility. Alternatively, as we found that knockdown of cohesin compromised HSV-1 replication compartment development and the phenotype is similar to what we observed in the HSV-1 replication with CTCF knockdown, cohesin could also exert supportive functions through genome organization during HSV-1 replication.

Importantly, cohesin can directly promote gene expression by regulating RNA polymerase II activity to target genes at the genome-wide scale [41]. The knockdown of cohesin component Rad21 led to the decreased transition of paused Pol II to elongation [41]. In KSHV, cohesin represses KSHV lytic gene expression during latency, and Rad21 depletion switched the paused form of RNA pol II to the elongation form of RNA pol II at the promoter of KSHV lytic gene ORF45 [16]. We previously reported that CTCF could promote HSV-1 transcription by facilitating the binding of CTD Serine 2 phosphorylated form of RNA Pol II and preventing the silencing of chromatin on the viral genome [34]. In this study, we found that cohesin knockdown resulted in decreased transcription of HSV-1 lytic genes, which is similar to the effect of CTCF on HSV-1 gene expression. We found a reduction in RNA Pol II recruitment and the accumulation of paused form of RNA Pol II, and an increase of silenced chromatin mark H3K27me3 binding to HSV-1 genes. Thus the facilitating role of cohesin in HSV-1 lytic infection differs from its role in KSHV and EBV. The difference could be because HSV-1 usually enters lytic infection in epithelial cells after primary infection, while KSHV tends to establish latent infection in B cells and endothelial cells. HSV-1 replicates rapidly in epithelial cells, so the experiments related to HSV-1 infection were mostly done shortly after infection. The lytic infection of KSHV may take several days, and large-scale chromatin conformation could play a bigger role.

Taken together, our finding that cohesin promotes HSV-1 replication and transcription broadens the understanding of how chromatin organizers play roles in the pathogen's life cycle and demonstrate the diversity of host protein functions in the interactions between virus and host.

## Conclusions

In this study, we found that cohesin subunits SMC1 and Rad21 were required for lytic HSV-1 replication. The depletion of cohesin results in decreased viral transcription, maturation of viral replication compartments, and viral reproduction. Cohesin prevents the recruitment of the pausing form of RNA polymerase II and the repressive chromatin marked by H3K27me3 to viral genes. These results suggest that cohesin facilitates HSV-1 lytic transcription by promoting RNA Pol II transcription activity and preventing chromatin's silencing on the viral genome.

## Methods

The methods were carried out under the approved guidelines.

### Cells and virus

BJ, HeLa, 293T, and Vero cells were originally obtained from American Type Culture Collection. Cells were maintained in DMEM (Gibco) containing 10% fetal bovine serum (FBS), penicillin (100 U/ml), and streptomycin (100 μg/ml) in a humidified 5% CO_2_ atmosphere at 37 ℃. HSV-1 17 + was kindly gifted by Dr. Chunfu Zheng from the Fujian Medical University. The virus was grown and titrated on Vero cells, as described previously [42]. Viral infections were done at indicated MOI. Briefly, the culture medium was replaced with serum-free DMEM, followed by adding the virus and incubating for 1 h, then the medium was replaced by regular DMEM with 10% FBS and 1% antibiotics. All experiments were carried out under the approved guidelines of the ethics committee of Kunming Institute of Zoology, and all experimental protocols were approved by the ethics committee of Kunming Institute of Zoology, Chinese Academy of Sciences.

### Antibodies

Monoclonal antibody against ICP4 is a gift from Gerd Maul's laboratory at the Wistar Institute [43, 44]. Antibodies against SMC1 (ab9262), SMC3 (ab9263), Rad21 (ab992), RNA Pol II (ab5408), RNA Pol II Ser5P (ab5131), H3K27me3 (ab6002) and IgG (ab46540) were from Abcam. CTCF polyclonal antibodies were bought from Abcam (ab70303), CTCF monoclonal antibodies were from Millipore (17-10044). Alexa Fluor 594 Goat Anti-Mouse IgG (H + L) Antibody and Alexa Fluor 488 Goat Anti-Rabbit IgG (H + L) Antibody were from Life Technologies.

### Immunofluorescence

Cells were seeded on glass coverslips in 24-well plates 24 h before infection and used for infections at indicated MOI. At 5.5 hpi, cells were fixed with 4% paraformaldehyde at 4 ℃ for 60 min and extracted with 0.2% Triton X-100 in PBS for 10 min. The nuclei were visualized by staining with Hoechst33342 and indicated proteins were stained by specific antibodies. Images were acquired using Nikon 80i. Images were taken with different channels for different samples. Images were merged and processed by Image J [45].

### SMC1, Rad21, and CTCF knockdown

For HeLa cells, siRNAs were used to knockdown indicated protein. Briefly, siRNAs GCAAUGCCCUUGUCUGUGAUU for SMC1, siRNAs AUACCUUCUUGCAGACUGU for Rad21, siRNAs GUAGAAGUCAGCAAAUUAA for CTCF were transfected into HeLa cells using Lipofectamine 2000 (Life Technologies, 11668019) according to the manufacturer's instructions. For BJ cells, shRNAs delivered by lentivirus were used to knockdown indicated protein. Briefly, shRNA CCGGGCCGGGACTGTATTCAGTATACTCGAGTATACTGAATACAGTCCCGGCTTTTTGAATTC for SMC1, shRNA CCGGGCTAATTGTTGACAGTGTCAACTCGAGTTGACACTGTCAACAATTAGC for ShRad21 were cloned into pLKO.1 vector. Furthermore, lentivirus was packaged by co-transfect pLKO.1 vector and pRSV-Rev, pMD2.G(VSV-G), pMDLg/pRRE package vector into HEK293T cells. Virion was collected from the medium by centrifuge at 48 h and 72 h after transfection and titrated by qPCR. HSV-1 infection was done after knockdown for 48–72 h.

### Isolation host and viral genomic DNA and RNA for qPCR and qRT-PCR analyses

Cells were infected with HSV-1 at an MOI of 5 and harvested at various time points. The genomic DNA was purified using the Genomic DNA purification kit (DP304-03, Tiangen). The RNA purification was purified using TRIzol (Ambion, 15596-018). Then, 1 μg RNA was reverse transcribed using Prime ScriptRT Reagent Kit with gDNA Eraser (TaKaRa, DRR047A) and stored at – 20 ℃. Real-time PCR was run in triplicate with 50 ng cDNA or 50 ng genomic DNA using FastStart Universal SYBR Green Master (Roche, 04913914001) ABI7900HT. Sequences of primers used are provided in the Additional file 1: Table S1. Viral DNA or RNA levels at each time point were quantified relative to the 0 hpi samples by the ΔCt method. To determine the relative DNA or RNA content at various times, average Ct valued for ICP0, ICP4, ICP8, and UL30 genes were subtracted by the average Ct values for 18 s. The calibrator value (HSV sample 0 hpi) was subtracted by the 18 s Ct value. To obtain the ΔΔCt value, the Ct value was subtracted by the Ct value of the input time point. ΔΔCt = (Ct_test_ − Ct_reference_)—(Ct_0 hpi sample_ − Ct_0 hpi 18 s_). The fold enrichment value is 2^−ΔΔCt^.

### Chromatin immunoprecipitation

ChIP assays were carried out according to the protocol from Chromatin Immunoprecipitation Assay Kit (Millipore) with minor modification. Briefly, cells were infected with HSV-1 at an MOI of 5. At 6 hpi, cells were fixed with formaldehyde (Sigma, final concentration 1% v/v). Then Glycine (125 mM) was added to stop the reaction. Cells were washed 3 times with ice-cold PBS then scraped from culture dishes into microfuge tubes. Cells were collected by centrifugation at 5000×*g* at 4 ℃ for 10 min. The cells were lysed by Lysis Buffer with protease inhibitors and sonicated to yield DNA fragments of between 200 and 500 bp in length. The samples were clarified by centrifugation at 13,000×*g* at 4 ℃ for 15 min, and the supernatant was diluted tenfold in IP Dilution Buffer with protease inhibitors. An aliquot (1/20) of each chromatin supernatant was reserved as the input sample. Dynabeads Protein G from INVITROGEN with a magnetic stand was used for immunoprecipitation. The chromatin supernatant was incubated with 5 μg antibody specific for total RNA pol II or RNA pol II ser 5 or H3K27me3 overnight at 4 ℃ with rotation. An aliquot was incubated with IgG (Abcam, ab2410) as a control to determine background binding. The beads were washed for 5 min at 4 ℃ with rotation, twice with Low-salt Buffer, once with High-salt Buffer, once with LiCl Buffer, twice with TE Buffer. Immunocomplexes were eluted by adding 210 μl of Elution Buffer incubating for 15 min at 65 ℃. Spin the beads at 13,000 rpm for 1 min and take 200 μl of the eluted solution and transfer to a new tube. Crosslinks were reversed by incubation for 7 h at 65 ℃ with a final concentration of 200 mM NaCl. The samples were then treated with RNase A and digested with proteinase K. DNA was purified by QIA quick PCR Purification Kit (QIAGEN, Cat. No 28104) and used as a template for real-time PCR.

## Supplementary Information

**Additional file 1: Table S1.** Primers for qPCR experiments. Sequences of primers used in qPCR experiments.

## Data Availability

Not applicable.

## Abbreviations

HSV-1: Herpes Simplex Virus type I
BJ cells: Human primary fibroblast cells
IF: Immunofluorescence
MOI: Multiplicity of infection
hpi: Hour post-infection
RT-qPCR: Quantitative reverse transcription PCR
ChIP: Chromatin immunoprecipitation
RNA Pol II: RNA polymerase II
RNA pol II ser5P: C-terminal domain serine 5 phosphorylated form of RNA polymerase II
H3K27me3: The 27th lysine residue tri-methylation form of the histone H3 protein
