# Supplementary material for: Cohesin promotes HSV-1 lytic transcription by facilitating the binding of RNA Pol II on viral genes
Source: Virol J. 2021 Jan 23;18:26. doi: 10.1186/s12985-021-01495-2 (PMC7825184; doi:10.1186/s12985-021-01495-2)
Supplement: Supplementary file 1 — Additional file 1: Table S1. Primers for qPCR experiments. Sequences of primers used in qPCR experiments. [file 12985_2021_1495_MOESM1_ESM.docx]

**Table S1. Primers for qPCR experiments.**

| Name | Sequence |
| --- | --- |
| ICP0 F | CTGCGCTGCGACACCTT |
| ICP0 R | CAATTGCATCCAGGTTTTCATG |
| ICP4 F | GCCCGGGCGCTGCTTGTTCTCC |
| ICP4 R | CGTCCGCCGTCGCAGCCGTATC |
| ICP8 F | GAGACCGGGGTTGGGGAATGAATC |
| ICP8 R | CCCCGGGGGTTGTCTGTGAAGG |
| UL30-F | TGTTTCGCGTGTGGGACATA |
| UL30-R | TTGTCCTTCAGGACGGCTTC |
| 18s F | GTAACCCGTTGAACCCCATT |
| 18s R | CCATCCAATCGGTAGTAGCG |
| ICP0 promoter F | CGCTTCCCGGTATGGTAATTAGAAAC |
| ICP0 promoter R | CGTGTGTTCCGCCAAAAAAGCAATTAGC |
| ICP4 promoter F | ATCGCATCGGAAAGGGACACG |
| ICP4 promoter R | CCAAGGTGCTTACCCGTGCAAA |
| ICP8/30 promoter F | GATCCGCCAGACAAACAAGG |
| ICP8/30 promoter R | AAAGAGGGGAATGTGGGGAG |
| UL36 promoter F | GGGTCTTAAGCACCTACGGA |
| UL36 promoter R | CCAACCACCCACCAAATAGC |
